# Synergistic modification of hot-melt extrusion and nobiletin on the multi-scale structures, interactions, thermal properties, and *in vitro* digestibility of rice starch

**DOI:** 10.3389/fnut.2024.1398380

**Published:** 2024-05-15

**Authors:** Zhihong Zhang, Ying Feng, Honglan Wang, Hai He

**Affiliations:** ^1^Department of Nutrition and Food Hygiene, School of Public Health, Heinz Mehlhorn Academician Workstation, Hainan Medical University, Haikou, Hainan, China; ^2^Department of Endocrinology and Metabolism, Shunde Hospital, Southern Medical University (The First People's Hospital of Shunde), Foshan, Guangdong, China

**Keywords:** rice starch–nobiletin complex, *in vitro* starch digestibility, thermal properties, multi-scale structure, hot-melt extrusion, molecular interaction

## Abstract

**Background:**

Rice starch has high digestibility due to its large carbohydrate content. Synergistic modification of hot-melt extrusion (HME) and additives such as flavonoids, hydrocolloids, proteins, lipids, and other additives has the tendency to retard the rate of starch hydrolysis. Hence, the current investigation aimed to study the combined effect of the HME-assisted addition of nobiletin (NOB, 0, 2, 4, and 6%) on the multi-scale structures, interactions, thermal, and digestibility characteristics of rice starch.

**Methods:**

The study employed density functional theory calculations and an infrared second derivative of an Fourier-transform infrared (FTIR) spectrometer to analyze the interactions between NOB and starch. The physicochemical properties of the starch extrudates were characterized by FTIR, ^13^C nuclear magnetic resonance, X-ray diffraction, and differential scanning calorimetry, while the digestibility was evaluated using an *in vitro* digestion model.

**Results:**

HME was found to disrupt the crystalline structure, helix structure, short-ordered structure, and thermal properties of starch. The interaction between NOB and starch involved hydrophobic interactions and hydrogen bonds, effectively preventing the molecular chains of starch from interacting with each other and disrupting their double helix structure. The addition of NOB led to the formation of a highly single-helical V-type crystalline structure, along with the formation of ordered structural domains. Consequently, the combined treatment significantly enhanced the ordered structure and thermal stability of starch, thus effectively leading to an increase in resistant starch and slowly digestion starch.

**Discussion:**

The study underscores that synergistic modification of HME and NOB holds promise for enhancing both the nutritional value and functional properties of rice starch. These findings offer valuable insights for developing high-quality rice starch products with broader applications.

## 1 Introduction

Rice is one of the most important staple foods in Asia, and the main component of rice, starch, provides most of the carbohydrates consumed by humans (1). Nonetheless, prolonged ingestion of rice starch-based goods has been strongly linked to elevated blood glucose levels after meals, which may lead to type 2 diabetes and further metabolic disorders (2). Therefore, it is important to find an appropriate modification to improve the digestibility of rice starch to meet the requirements of low glycemic index (GI) value rice foods. The emphasis on modifying starch digestibility has increased recently. These methods include processing food to increase the amount of resistant starch (RS), physically changing the starch’s molecular structure, or adding non-starch ingredients like proteins, lipids, flavonoids, and others to interact molecularly with starch and change its physicochemical and digestibility properties (3, 4).

Hot-melt extrusion (HME) technology could provide a lead in the restructuring of heterogeneous food matrix at the molecular level because of the processing capabilities of extruders. These capabilities encompass simultaneous processes such as flavor generation, encapsulation, heating, cooling, shaping, venting, mixing, shearing, and conveying. The versatility of HME technology allows for efficient and comprehensive transformations of diverse dietary matrices (5). Additionally, HME may result in improved water hydration capabilities, degree of soluble fiber content from cell wall components, digestibility, shelf-stability, and expansion ratio (6). During the HME process, food ingredients such as proteins, lipids, hydrocolloids, bioactive compounds, fibers, and others may be added to the extruded food to enhance its quality (7). Utilizing extruders as a bioreactor, starch molecules are esterified with organic acids to create cross-linked starch with a higher RS content (8). During processing, food ingredients undergo physicochemical and structural changes that are often intended to enhance the product’s digestibility, nutritional bioavailability, textural, organoleptic, sensory requirements, and storage stability (9).

Phenolic compounds like flavonoids and tannins can form non-covalent bonds with starch. These bonds affect starch’s structural and functional characteristics, including hydrogen bonds, hydrophobic interactions, and van der Waals forces (10–12). Previous studies indicate that inclusion (V-type) and non-inclusion complexes may be formed by interacting starches with various phenolic chemicals (10–12). The V-type inclusion complex was effectively embedded within the internal hydrophobic helix of the starch, achieving successful encapsulation. For example, A classic V-type diffraction peak was seen at 2θ = 13.0° and 19.8° in the inclusion complex created by gallic acid and rice starch (13). The starch-lipid complex (RS_5_), which belongs to the RS_5_ family, exhibits a similar organizational mechanism to that of the inclusion complex formed by starch-polyphenol compounds (14). As previously indicated, non-covalent connections between polyphenol chemicals and starch facilitate direct interactions, which are specifically referred to as the non-inclusion complex. However, this is not the same as the V-type inclusion complicated development (15). For instance, in a study conducted by Huang et al. (16), it was found that lotus seed starch and tea polyphenol formed a non-inclusion complex following treatment with high hydrostatic pressing. This was confirmed by inspection using confocal laser scanning microscopy and scanning electron microscopy.

Nobiletin (NOB) is an O-methylated flavonoid widely found in citrus peels, and is structurally characterized by the presence of a benzopyran moiety and multiple methoxyl groups on the aromatic ring (17). This combination of hydroxyl and methoxy groups modifies the structural and functional characteristics of starch and suppresses the activity of amylase (18). In our current study, we employed physical modification through HME to craft the final product with altered characteristics. However, the study on the production of starch–NOB complex with high enzymatic digestion resistance under HME has been limited, and the starch–NOB complex interaction has not been fully explained. Therefore, the objective of this study was to investigate the effect of HME with different NOB concentrations on the multi-scale structures, interactions, and thermal and digestibility properties of rice starch. This work could guide industrially HME production of digestion-resistant starches derived from the starch–flavonoids complex.

## 2 Materials and methods

### 2.1 Reagents

The rice starch (Remy-DR) with 64.10% amylopectin, 22.45% amylose, 10.25% (d.b) moisture, 0.48% (d.b) protein, 0.20% (d.b) lipid, and 0.24% (d.b) ash was supplied by Beneo-orafti (Oreye, Belgium). Shanghai, China-based Yuanye Bio-Technology Co., Ltd. provided the NOB. Sigma-Aldrich Co., Ltd. (Sigma, St Louis, MO, United States) provided the amyloglucosidase (A3306, activity 318 U/mL) and porcine pancreatic ɑ-amylase (P7545, activity 8 × USP). Megazyme (Wicklow, Ireland) provided the glucose oxidase-peroxidase (GOPOD) test kit. Analytical reagents were all other substances.

### 2.2 Twin-screw extruder-based in-situ complexation of rice starch with NOB

Based on rice starch, various NOB concentrations (0, 2, 4, and 6% w/w) were added to 40% moisture-content rice starch. The mixture was homogeneously blended using a mixing agitator at 100 r/min for 5 min. Next, the reaction mixtures were introduced into a co-rotating twin-screw extruder with a 52 length-to-diameter ratio (HK-36 model, Nanjing KY Chemical Machinery CO., Ltd., Nanjing, China). The extruder operated at a screw speed of 90 r/min and maintained a heating temperature of 85°C. This procedure facilitated the homogenization and gelatinization of the mixtures, producing a uniform and consistent product. The gelatinization of the mixture is further facilitated by the high shear stress of the extruder (19). Following a 48-h drying procedure at 45°C, the extrudates were processed in a room-temperature mill (BJ-800A, Baijie, Huzhou, China). After passing through a 100 mesh sieve, the powder samples were stored at room temperature with a moisture content of less than 8% and kept out of direct sunlight.

### 2.3 Analysis of the starch digestibility *in vitro*

The *in vitro* digestibility of the NRS and HMERS samples was evaluated following with the previous studies (20, 21). 1 g of dry-based starch was placed in sodium acetate buffer (20 mL, 0. 1 M, pH = 5. 2), and the starch samples were hydrolyzed with an enzyme mixture (0. 5 mL, 190 r/min, 37°C) containing porcine pancreatic α-amylase (300 U/mL) and amyloglucosidase (20 U/mL). When the *in vitro* digestion process was underway, 0.5 mL of digestible fluid was taken at 0, 20, 60, 120, and 180 min. Each sample received 25 mL of 70% ethanol to fully inactivate the enzyme. The samples were then centrifuged (4,000 r/min, 5 min), and the supernatant (0.1 mL) was added to GOPOD (3 mL). Following that, the samples were incubated for 20 min at 45°C. Eqs. 1–4 were used to compute the quantities of rapidly digested starch (RDS), slowly digested starch (SDS), and RS, taking into account the values of G_20_ and G_120_.


(1) $$\text{C (\%) = }\frac{G_{t}-G_{0}}{TS}\text{ × 0.9 × 100}$$

(2) $$\text{RDS (\%) = }\frac{G_{20}-G_{0}}{TS}\text{ × 0.9 × 100}$$

(3) $$\text{SDS (\%) = }\frac{G_{120}-G_{0}}{TS}\text{ × 0.9 × 100}$$

(4) $$\text{RS (\%) = }\frac{TS-\left(RDS+SDS\right)}{TS}\text{ × 100}$$

t stands for the digestion time points, while C (%) is the starch digestibility rate. G_t_ represents the amount of glucose present at time t, whereas G_0_, G_20_, and G_120_ stand for the amounts present at 0, 20, and 120 min, respectively. TS stands for total starch samples.

To evaluate starch samples’ *in vitro* digestibility and forecast their physiological reaction, we used the logarithm of the slope (LOS) (22, 23). First-order kinetics adapts to digestibility in the following way Eq. 5:


(5) $$C_{t}=C_{\infty }\left(1-e^{-kt}\right)$$

C_t_ represents the starch sample’s digested ratio at time t (min) and C_∞_ indicates the digested ratio at the reaction’s ending. K stands for the first-order kinetics rate constant. We generated a LOS plot by differentiating the first-order equation and then representing it in a logarithmic format (Eq. 6):


(6) $$\ln\left(\frac{dC}{dt}\right)=-kt+\ln\left(C_{\infty }k\right)$$

### 2.4 Predicted glycemic index (pGI)

To assess the hydrolysis index (HI), the starch digestion curve was obtained at different time intervals (0, 20, 60, 120, and 180 min). The HI was determined by calculating the proportion of total glucose released within 180 min compared to the glucose released from white bread within the same time period. To estimate the pGI, the formula (pGI = 44.78 + 0.3797 × HI) established by Goni et al. (24) with slight modifications was utilized.

### 2.5 Differential scanning calorimetry (DSC)

To examine the thermal properties of the NRS and HME samples, the DSC (DSC-1, Mettler Toledo, Zurich, Switzerland) was used. Weigh the sample in an aluminum pot (keeping an empty pot as a reference) to a weight of about 6 mg. Before measurement, the temperature was calibrated using indium at the same scanning rate. Dry nitrogen (50 mL/min) was used to scan the samples at 30–120°C (10°C/min).

### 2.6 Analysis of the complex’s multi-scale structure

The molecular interactions of the NRS and HMERS samples were investigated using an attenuated total reflectance (ATR) Fourier-transform infrared spectrometer (FTIR) equipped with a deuterated triglycine sulfate detector (Nicolet iN10, Thermo Fisher Scientific, United States). Sixty-four scans were carried out at a resolution of 4 cm^−1^ in the 4,000–400 cm^−1^ range. The content of the ordered structure with a short range was indicated by the absorption ratios (*R*_1047/1022_) at 1,047 and 1,022 cm^−1^ (25).

^13^C CP/MAS NMR (cross-polarization magic angle spinning carbon-13 nuclear magnetic resonance) spectroscopy and a magnet 400 (Bruker, Karlsruhe, Germany) were used to examine the helical structures of the NRS and HMERS samples. Before testing, the moisture concentrations of the complex were adjusted. A 4-mm MAS solid-state probe was used for the ^13^C CP/MAS NMR test, which was conducted at a frequency of 150.9 MHz. 10,000 kHz was the speed at which the test was carried out, along with 90° (5 μs) pulse width, 1 ms exposure period, 5 s delay, and at least 2,400 total frequency cycles. The temperature during the test was maintained at 25°C. The spectra were assessed, and the contents of the helix structure were ascertained using PeakFit v4.12 (26).

The NRS and HMERS samples’ X-ray diffraction (XRD) patterns were examined using an advanced wide-angle XRD (D8, Bruker, Germany), according to a previously defined procedure (26). The diffraction strengths (2θ) of NRS and HMERS samples were scanned from 5° to 60° (40 kV, 30 mA, scan speed = 2°/min, step size = 0.013°). The proportions of A-type (%) and V-type (%) were calculated by an MDI Jade program (Materials Data, Livermore, CA, United States, V6.5) in the range of 5°–40° (2θ), and the total crystalline (*X*_Total_, %) was computed as = A-type (*X*_A_, %) + V-type (*X*_V_, %) (26).

### 2.7 Computational method for the interactions of starch and NOB

Gaussian View software was used to generate the initial structures of the rice starch–NOB complex, rice starch, and NOB for density functional theory (DFT) computations. The optimized molecular structures and corresponding vibrational assignments of rice starch, NOB, and the complex have been investigated by using DFT B3lyp/6-31 g(d) (27, 28). Atoms-in-molecule theory (AIM) analyses were performed using the Multiwfn program (29) to provide more information on the interactions of the complex. Gaussian 09 software was used for all other computations (30).

### 2.8 Analytical statistics

The mean ± the standard deviation (SD) was used to represent the experimental data. ANOVA and Duncan’s multiple range test were used to find any differences in the data analysis, which was done with SPSS software (V22.0, Inc. Chicago, IL, United States). Significant was defined as *p* < 0.05.

## 3 Results and discussion

### 3.1 *In vitro* digestibility

NRS had 1.44% RS, 3.52% SDS, and 95.04% RDS, as shown in Table 1. The increase in the quantities of SDS and RS in HMERS (*p* < 0.05), coupled with a decrease in the quantity of RDS in the sample (*p* < 0.05), suggests that the HME method employed in this study attenuated the digestion of NRS. Shear-induced starch molecule fragmentation that leads to increased retrogradation during storage is the cause of this decline (31). Additionally, there was a significant increase in the content of NOB and a considerable rise in the content of SDS and RS. An increase in SDS content from 6.62 to 8.33% (*p* < 0.05), and the RS content rose from 11.16 to 19.85% (*p* < 0.05). Together, these findings indicate that the binding of NOB leads to an increase in RS content. The strong crystalline structure that results from the interaction of NOB and rice starch is partly responsible for the increase in resistance to enzymatic hydrolysis. Furthermore, the resistance to enzymatic hydrolysis is further enhanced by NOB via hydrophobic contacts and hydrogen bonding with the active region of α-amylase (32). In line with the findings of Lemlioglu-Austin et al. (33), tannin was observed to decrease starch digestibility when combined with starch, resulting in a reduced pGI. Our experiments similarly showed that interactions with NOB led to a significant decrease in the pGI of HMERS (*p* < 0.05). In addition, the pGI of HMERS decreased gradually with increasing NOB content (Table 1).

**Table 1:** Evaluation of RDS, SDS and RS content, digestibility parameters of LOS plots, and pGI for NRS and HMERS samples.^*^

Samples	k (min^−1^)	C_∞_(%)	RDS (%)	SDS (%)	RS (%)	pGI
NRS	0.1765 ± 0.0032^e^	97.536 ± 0.680^e^	95.04 ± 0.19^e^	3.52 ± 0.20^a^	1.44 ± 0.55^a^	91.87 ± 1.02^d^
HMERS	0.1447 ± 0.0021^d^	91.768 ± 0.392^d^	86.74 ± 0.15^d^	5.33 ± 0.34^b^	7.93 ± 0.43^b^	81.25 ± 1.05^c^
HMERS/NOB-2%	0.1348 ± 0.0030^c^	88.283 ± 0.645^c^	82.22 ± 0.32^c^	6.62 ± 0.26^b^	11.16 ± 0.22^c^	80.19 ± 0.93^b^
HMERS/NOB-4%	0.1248 ± 0.0021^b^	87.495 ± 0.301^b^	80.51 ± 0.22^b^	7.43 ± 0.26^c^	12.06 ± 0.32^d^	79.39 ± 0.80^b^
HMERS/NOB-6%	0.1158 ± 0.0020^a^	79.515 ± 0.754^a^	71.82 ± 0.25^a^	8.33 ± 0.30^d^	19.85 ± 0.36^e^	76.71 ± 1.10^a^

^*^Results are means ± standard deviation of three replicate tests (*n* = 3); values indicated by an additional letter are regarded as *p* < 0.05.C_∞_, the equivalent value at the endpoint; k (min^−1^), the digestion rate constant; RDS, rapidly digestion starch; SDS, slowly digestion starch; RS, resistant starch; pGI, predicted glycemic index; NRS, native rice starch; LOS, the logarithm of the slope; HMERS, hot-melt extruded rice starch; HMERS/NOB, hot-melt extruded rice starch–nobiletin complex.

### 3.2 Analysis of the first-order kinetics

Supplementary Figure 1 displays the digestibility plots and LOS curves for the NRS and HMERS samples. All graphs clearly show an exponential growth shape, characterized by a rapid increase period, followed by a slow digestion phase until the maximum digestion range of starch was reached and digested after 120 min. According to this result, first-order kinetics were likely followed in the digestion of the NRS and HMERS samples. The k and C_∞_ values that were taken from the samples’ LOS curves throughout the digestion process are shown in Table 1. The results showed that a single digestion phase (*R*^2^ > 0.99) effectively replicated the entire digestive process, indicating comparable α-amylase contents in both NRS and HMERS samples (34). In this regard, the substantial variations in the k and C_∞_ values between the NRS and HMERS samples were corroborated by our findings. The values of k and C_∞_ for NRS were 0.1765 min^−1^ and 97.536%, while HMERS had values of 0.1447 min^−1^ and 91.768% (*p* < 0.05). In the HMERS/NOB samples, the presence of NOB led to a reduction in glucose release rate. Overall, these findings reflect a consistent pattern with the digestibility profile of the starch samples outlined in Table 1.

### 3.3 Thermal properties

The DSC data (Supplementary Figure 2 and Table 2) reveals that the HME treatment significantly reduced the onset temperature (T_o_), peak temperature (T_p_), and conclusion temperature (T_c_) of NRS compared to its pre-HME treatment state. The low gelatinization state of NRS after HME treatment indicated the structural instability of starch granules due to the low ordering and crystallinity (35). In addition, the NRS after HME treatment showed a significant reduction in enthalpy (ΔHg) compared to the NRS before (*p* < 0.05). This can be attributed to the decreased double helix structure observed in the HME samples. Table 2 show that T_o_, T_p_, T_c_, and ΔHg increased with the addition of NOB (*p* < 0.05). The increase in these values indicates an improvement in the crystalline homogeneity and crystalline structure of the starch. As the crystalline structure increased, the energy required to destroy the crystalline structure increased (36), resulting in a higher gelatinization ΔHg of the starch after NOB addition.

**Table 2:** Thermal properties for NRS and HMERS samples.^*^

Samples	T_o_ (°C)	T_p_ (°C)	T_c_ (°C)	ΔHg (J/g)
NRS	59.1 ± 0.3^e^	82.2 ± 0.1^d^	95.4 ± 0.2^e^	1.912 ± 0.012^e^
HMERS	46.5 ± 0.2^a^	62.1 ± 0.2^a^	73.3 ± 0.3^a^	0.582 ± 0.015^b^
HMERS/NOB-2%	48.3 ± 0.1^b^	62.5 ± 0.2^a^	74.5 ± 0.1^b^	0.472 ± 0.015^a^
HMERS/NOB-4%	49.6 ± 0.2^c^	63.4 ± 0.1^b^	75.7 ± 0.3^c^	1.001 ± 0.013^c^
HMERS/NOB-6%	53.6 ± 0.3^d^	65.1 ± 0.2^c^	78.9 ± 0.1^d^	1.371 ± 0.013^d^

^*^Results are means ± standard deviation of three replicate tests (*n* = 3); values indicated by an additional letter are regarded as *p* < 0.05.T_o_, T_p_, T_c_, and ΔHg are the onset temperature, peak temperature, conclusion temperature, and enthalpy. NRS, native rice starch; HMERS, hot-melt extruded rice starch; HMERS/NOB, hot-melt extruded rice starch–nobiletin complex.

### 3.4 Short-range order structure, helical structure, and crystalline structure

**Figure 1:**
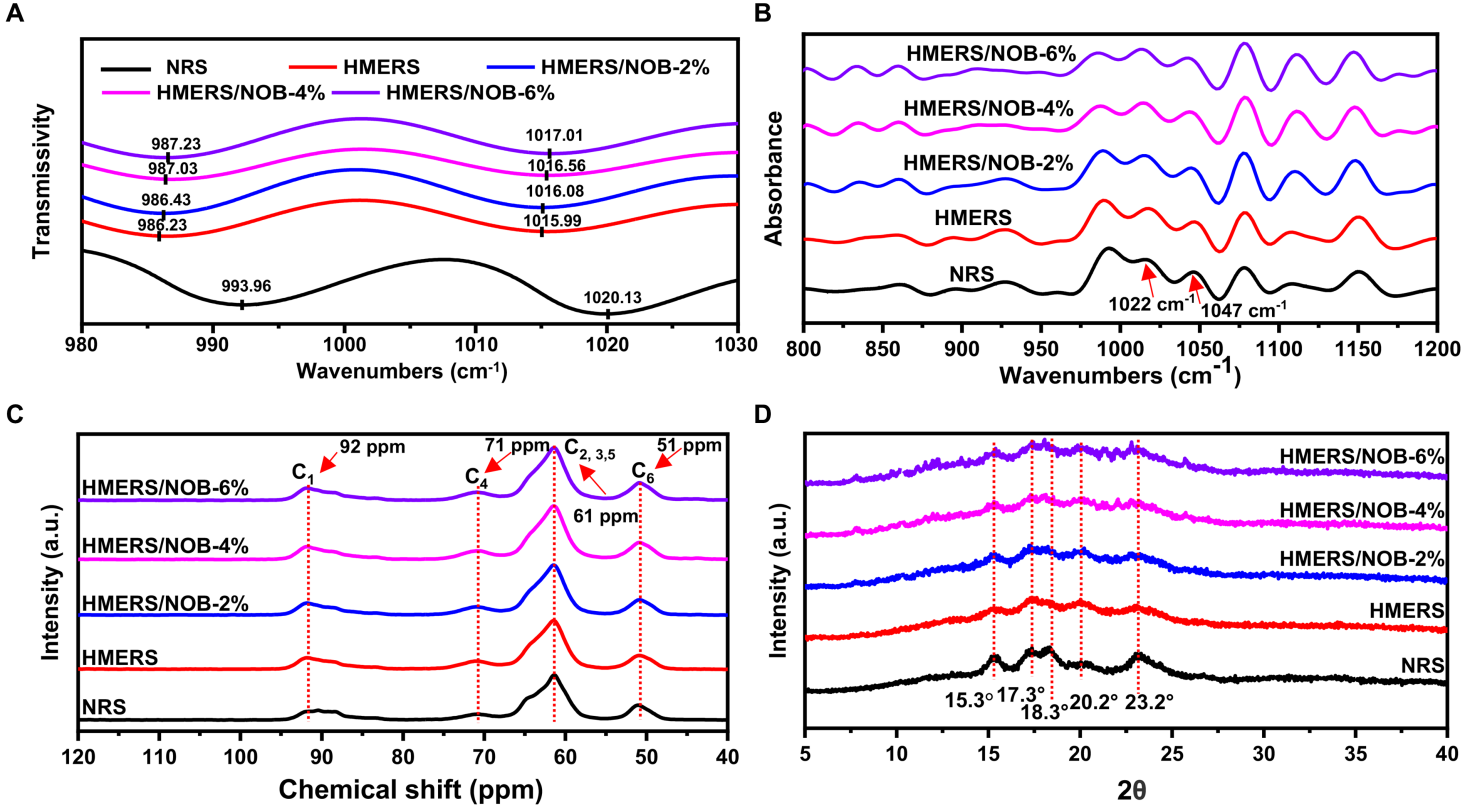
**(A)** Infrared spectroscopy with second derivative, **(B)** spectra of deconvoluted ATR-FITR, **(C)** ^13^C NMR spectra, and **(D)** XRD spectra for NRS and HMERS samples. NRS, native rice starch; HMERS, hot-melt extruded rice starch; HMERS/NOB, hot-melt extruded rice starch–nobiletin complex.

The vibrational absorption peaks at 1022 and 997 cm^−1^ correspond to C-O-H bonds. The former is associated with the chain structure of the starch molecule, while the latter is linked to the intramolecular hydrogen bonding of the hydroxyl group on the dehydrated glucose unit C_6_ of the starch molecule (37). Since it reflected more chemical structure details and had a better resolution, the second-order infrared spectrum and the starch samples’ infrared spectra were analyzed using the second-order mode, as Figure 1A illustrates. When comparing the NRS spectra, the low-frequency peaks in HMERS were found to be moving at 1022 and 997 cm^−1^ (Table 3). This change may be ascribed to the mechanical contact between heat energy and water molecules during the HME process, which breaks the starch molecular chain and causes it to lose its double-helical structure. Consequently, there is an increase in the amount of amorphous structure, which makes the starch molecule chain easier to move and reassemble. Ultimately, this causes the starch molecules to form hydrogen bonds with each other (38). The addition of NOB shifted towards a higher frequency of the infrared absorption maxima located at 1022 and 997 cm^−1^ (Table 3). The absorption bands observed at 1022 and 997 cm^−1^, primarily attributed to C-O-H bending vibrations, exhibit heightened sensitivity to variations in water content. These vibrations, such as hydrogen bonding, are likely influenced by interactions between water and starch molecules, consequently affecting the C-O-H bending modes. Alterations in this spectral region have been attributed to fluctuations in the molecular environment surrounding the primary hydroxyl group in V-type amylose, stemming from shifts in intramolecular hydrogen bonding dynamics (39). Hydrogen bonding between starch and NOB and between starch molecules was revealed by the HME treatment. This resulted in the formation of a spatially ordered aggregate, which decreased the amount of amorphous HMERS structure.

**Table 3:** The parameters being analyzed for NRS and HMERS samples include helical structures, total crystallinity, A-type crystallinity, V-type crystallinity, short-range ordered degree, C-O-H def., and CH_2_.^*^

Samples	Amorphous	Single helix	Double helix	*X*_Total_ (%)	*X*_A_ (%)	*X*_V_ (%)	*R*_1047/1022_	C-O-H def., CH_2_	
NRS	55.21 ± 0.13^a^	2.33 ± 0.17^a^	42.46 ± 0.16^e^	25.43 ± 0.15^d^	24.91 ± 0.14^e^	0.52 ± 0.10^a^	0.455 ± 0.013^a^	1020.13	993.96
HMERS	78.61 ± 0.12^e^	3.18 ± 0.19^b^	18.21 ± 0.25^d^	11.18 ± 0.18^a^	9.49 ± 0.13^d^	1.69 ± 0.05^b^	0.566 ± 0.006^b^	1015.99	986.23
HMERS/NOB-2%	74.19 ± 0.14^d^	9.55 ± 0.23^c^	16.26 ± 0.23^c^	11.46 ± 0.20^a^	8.13 ± 0.11^c^	3.33 ± 0.09^c^	0.579 ± 0.003^c^	1016.08	986.43
HMERS/NOB-4%	72.32 ± 0.20^c^	14.33 ± 0.32^d^	13.35 ± 0.23^b^	12.26 ± 0.22^b^	7.78 ± 0.12^b^	4.48 ± 0.10^d^	0.591 ± 0.002^d^	1016.56	987.03
HMERS/NOB-6%	70.53 ± 0.30^b^	17.58 ± 0.36^e^	11.69 ± 0.15^a^	13.18 ± 0.10^c^	6.64 ± 0.10^a^	6.54 ± 0.09^e^	0.623 ± 0.005^e^	1017.01	987.23

^*^Results are means ± standard deviation of three replicate tests (*n* = 3); values indicated by an additional letter are regarded as *p* < 0.05.*X*_A_, A-type crystallinity; *X*_V_, V-type crystallinity; *X*_Total_, total crystallinity; *R*_1047/1022_, short-range ordered degree; C-O-H def., CH_2,_ the wavenumbers of C-O-H bonds; NRS, native rice starch; HMERS, hot-melt extruded rice starch; HMERS/NOB, hot-melt extruded rice starch–nobiletin complex.

The short-range ordered structure of starch is visible in the infrared spectrum at an absorption ratio of 1047/1022 cm^−1^ (*R*_1047/1022_) (40, 41). Figure 1B displays the starch samples’ deconvoluted FTIR spectra in the 1,200–800 cm^−1^ range, and the *R*_1047/1022_ values are compiled in Table 3. Before the HME procedure, the *R*_1047/1022_ value was 0.455, but after the HME treatment, the NRS value climbed to 0.566 (*p* < 0.05). Comparable results were obtained with the HMERS/NOB samples. NOB addition could effectively improve R_1047/1022_ as indicated by increased short-range order degree of NOB-treated rice starch. The degree of order in the starch molecules was enhanced by creating strong hydrogen bonds between the –O–H proton and the –C–H proton of the benzene ring on NOB (42). Additionally, the *R*_1047/1022_ of HMERS/NOB samples were slightly increased with NOB addition because NOB was easily inserted into the single helical cavity of starch and formed a V-type inclusion complex (42). Meanwhile, NOB formed a non-inclusion complex with starch molecules through hydrogen bonding (43), producing relatively dense local-order structures.

^13^C CP/MAS NMR spectroscopy was used to evaluate the starch samples’ helical structures. According to prior research, changes in starch’s helical structures may affect chemical shifts in starch and glucose caused by C_1_ and C_4_ (Figure 1C) (44). The contents of single and double helical structures found in starch samples are shown in Table 3. Following the HME process, HMERS’s single helix structure content rose from 3.18% to 2.33% (*p* < 0.05), while the percentage of its double helix structure content dropped from 42.46% to 18.21% (*p* < 0.05). Thermal processing breaks the α-1,4 and α-1,6 glycosidic bonds, increasing the amount of straight-chain starch and creating more single helix structures (45, 46). The susceptibility to shear degradation and branch density increase with shorter starch branch lengths (47). After NOB addition, we observed an increase in the single helical structures (9.55% to 17.58%, *p* < 0.05) and a decrease in the double helical structure content (16.26% to 11.69%, *p* < 0.05) in the HMERS/NOB samples. The strong hydrogen bonding between starch and NOB may be responsible for the slower binding of the helical structures, which is one of the reasons for the reduction of the double helix structures (48).

The XRD patterns displaying the broad angles for the starch samples are depicted in Figure 1D. At 15.3°, 17.3°, 18.3°, and 23.2° (2θ), the NRS exhibited characteristic peaks that are indicative of a particular A-type crystalline structure (49). Because of the differences in the XRD spectrum of HMERS and NRS, as well as the larger peak shapes and lower peak intensities, it was concluded that the HME treatment had a negative impact on the A-type crystalline structure of NRS. Furthermore, the HME treatment resulted in a considerable reduction in the relative degree of crystallinity and a reduction in the double helix structure content found in rice starch (Table 3). Furthermore, a notable diffraction peak at 20.2° (2θ) was seen by HMERS, encouraging the development of a crystalline structure in the V-type. The crystalline structure of the HMERS/NOB samples, characterized by an A + V-type arrangement, is evident from the peaks observed in the spectra at 15.3°, 17.3°, 18.3°, 20.2° and 23.2°(2θ) (50). The addition of NOB to rice starch increased the intensity of the V-type crystallization peaks and decreased the intensity of the A-type crystallization peaks. Table 3 shows that the A-type content decreases from 8.13% to 6.64% with the addition of NOB (*p* < 0.05), while the V-type content increases from 3.33% to 6.54% (*p* < 0.05). The A-type and V-type crystalline structures are derived from the double and single helical structures of starch (42). These results matched the ^13^C NMR-characterized helical structures rather well (Table 3).

### 3.5 Interactions between NOB and rice starch

**Figure 2:**
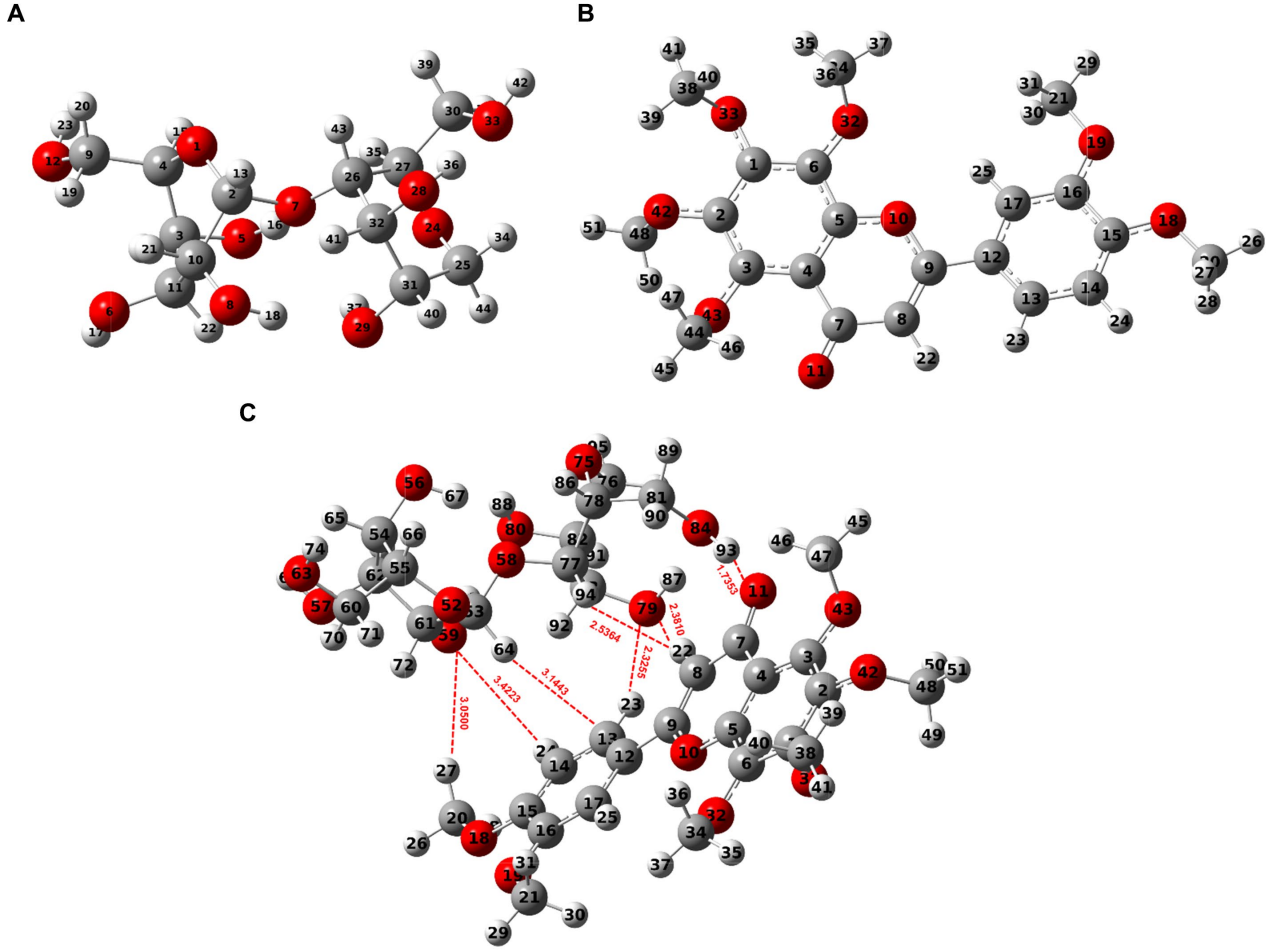
Optimized stable conformer of glucose dimer-NOB system [DFT B3lyp/6-31 g(d)]. **(A)** Glucose dimer (Starch). **(B)** NOB. **(C)** Glucose dimer + NOB. The O atom is represented by the red sphere, the C atom by the grey sphere, and the H atom by the white ball. A dotted red line indicates hydrogen bonds. NOB, nobiletin.

The findings above showed that the dynamic interactions between starch molecules and NOB during HME were responsible for the multi-scale structural alterations in HMERS/NOB. The intermolecular interaction location and intensity of the HMERS/NOB samples were investigated using further DFT computations. We first perform a geometric structure optimization to simulate the physical molecular interactions between starch and NOB using DFT calculations. Figure 2 shows the optimized structures of glucose dimer (starch) (51), NOB, and rice starch–NOB complex formed from glucose dimer and NOB. Based on previous studies, we calculated the typical configuration of glucose dimer–NOB complex (52, 53). As shown in Figure 2C and Table 4, the Ḥ··O bond lengths in the system are in the range of 1.7353–3.4223 Å, which can be assigned to hydrogen bond interactions between glucose dimer and flavones (54). The number of hydrogen bonds was seven for the glucose dimer and NOB system. Therefore, hydrogen bonding exists between glucose dimer and NOB. Specifically, for glucose dimer to form hydrogen bonds, the active sites as donors were oxygen atoms of hydroxyl oxygen on the 59(O) and 79(O), and hydrogen atoms as acceptors are hydrogen atoms of 64(H), 93(H), and 94(H) (Figure 2C). For NOB, the active sites as donors were oxygen atoms of hydroxyl oxygen on the 11(O) and 13(C), and hydrogen atoms as acceptors are hydrogen atoms of 22(H), 23(H), 24(H), and 27(H) (Figure 2C).

**Table 4:** The characteristics of the BCP (a.u.), the lengths of the hydrogen bonds (Å), and the energies of the hydrogen bond interactions for the glucose dimer + NOB.^*^

Glucose dimer + NOB					
Interaction modles	HB length (Å)	*ρ*_BCP_	∇^2^*ρ*_BCP_	H_BCP_	|ΔE(H)| (kcal/mol)
93(H)-11(O)	1.7353	0.0432	0.1299	−0.0018	8.8948
94(H)-22(H)	2.5364	0.0041	0.0147	0.0010	0.1723
79(O)-22(H)	2.3810	0.0125	0.0379	0.0002	2.0462
79(O)-23(H)	2.3255	0.0137	0.0438	0.0002	2.3139
64(H)-13(C)	3.1443	0.0037	0.0106	0.0006	0.0831
59(O)-24(H)	3.4223	0.0015	0.0071	0.0005	0.4077
59(O)-27(H)	3.0500	0.0025	0.0106	0.0007	0.1846

^*^ρ_BCP_, electron density; ∇^2^*ρ*_BCP_, the laplacian value of electron density; H_BCP_, the energy density of electrons at the BCP point; |ΔE(H)|, the energies of the hydrogen bond interactions; NOB, nobiletin.

The AIM provides a general tool for classifying binding interactions (55). According to AIM topology analysis, charge density (*ρ*_BCP_) is used to characterize the bond strength. In general, the higher the value of *ρ*_BCP_, the stronger the bonding. The value of Laplacian (∇^2^*ρ*_BCP_) indicates the type of interaction between atoms. A negative value (∇^2^*ρ*_BCP_ < 0) suggests the presence of covalent bonds, while a positive value (∇^2^*ρ*_BCP_ > 0) indicates that the interaction between atoms is primarily governed by electrostatic interactions (55). Furthermore, the binding interactions may be more accurately described by the energy density (H_BCP_) of electrons at the bond critical point (BCP) (56, 57). When H_BCP_ > 0, then ∇^2^*ρ*_BCP_ > 0 > 0, indicating that the interaction between the two atoms is dominated by electrostatic interactions; if H_BCP_ < 0, then two scenarios occur: (1) ∇^2^*ρ*_BCP_ < 0, indicating that the interaction is dominated by covalent bonding interactions; and (2) ∇^2^*ρ*_BCP_ > 0, indicating that the interaction is dominated by electrostatic interactions, but already contains some covalent bonding components. The values of the *ρ*_BCP_ and ∇^2^*ρ*_BCP_ are distributed in the range of 0.0015–0.0432 a.u. and 0.0106–0.1299 a.u., with H_BCP_ > 0 [except for 93(H)-11(O)] (Table 4), indicating a predominant reliance on hydrogen bonding to evaluate the complex interactions (58). By employing the empirical hydrogen bond energy formula, it is possible to calculate the binding energy of an intermolecular hydrogen bond: ΔE(H) ≈ −223.08 × *ρ*_BCP_ + 0.7423 (58). The sum energies of the hydrogen bonds were found to be 14.1026 kcal/mol for the complex of glucose dimer–NOB. These findings indicate the strength of hydrogen bond interactions between NOB and glucose dimers.

### 3.6 Regulation mechanism of HME with NOB on digestibility of rice starch

**Figure 3:**
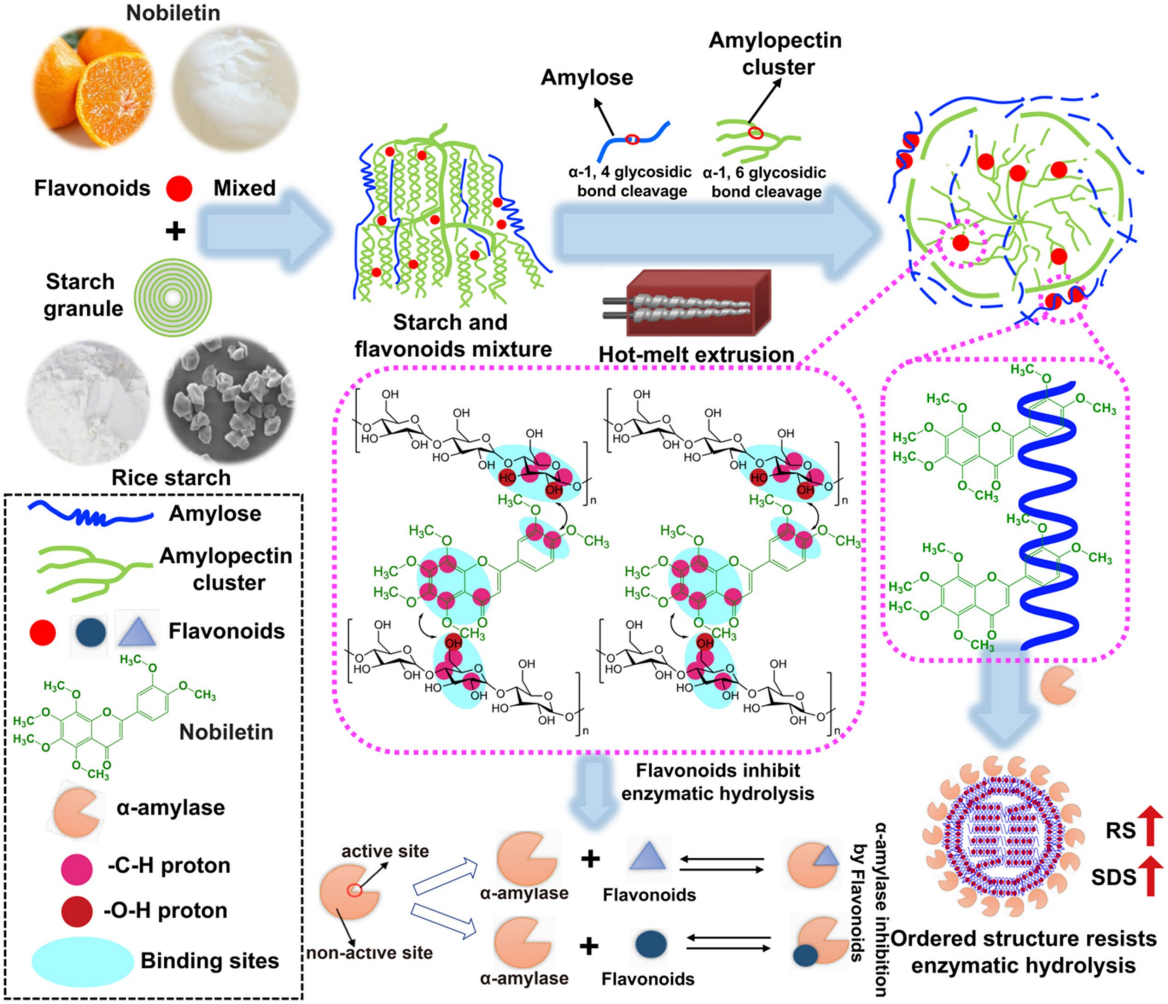
Schematic diagram of a model of synergistic modification of HME and NOB to regulate the digestibility and multi-scale structure of rice starch. Magenta circle, -C-H proton; Red circle, -O-H proton; Cyan circle, hydrogen bond position; Large circle, strong correlation of binding sites; Small circle, weak binding site correlation. HME, hot-melt extrusion; NOB, nobiletin.

Based on the digestibility, thermal properties, multi-scale structure, and theoretical calculations of the above HMERS/NOB samples, Figure 3 depicts the molecular mechanism of NOB in regulating rice starch digestibility during the HME process. When heat energy, shear stress, and cooperative water molecules worked together, they disrupted the α-1, 4 and α-1, 6-glycosidic bonds in rice starch, lowering the molecular weight (38). Hydrogen bond interactions between rice starch molecules and water molecules also resulted in molecular chain depolymerization, the dissolution of the crystalline structures, and the disintegration of the double helix structures (Table 3). Conversely, HME promoted the rearrangement, agglomeration, and synthesis of the starch molecular chain, creating a novel single helical structure, a V-type crystal structure including the lipid within the starch, and a short-range ordered structure (Table 3). These dense domains with a local order hindered the migration of α-amylase in starch molecules and obstructed α-amylase interaction sites. This in turn reduces the RDS content and increases the SDS and RS content in rice starch, thereby enhancing the slow digestibility and resistance to digestion of rice starch (Table 1).

Under HME, the occurrence of intramolecular hydrogen bonding leads to the rotation of the starch chain, resulting in the formation of a left-handed spiral hollow structure (59). When NOB became available, V-type crystalline was formed via hydrophobic interactions in helical cavities. The complex was simultaneously stabilized by hydrogen bonding between the –O–H protons on the starch glucose unit and the –C–H protons of the benzene ring on the NOB (Figure 3). In addition to the common –C–H donor groups found in alkyl and aromatic compounds, various chemicals form unique bonds with specific starch molecules through two or three weak CH-π and hydrogen interactions (Figure 2 and Table 4) (60). The above effects strengthen the structural order of the complex in terms of both short-term and long-term structures (Table 3). Overall, the multi-scale structure of the HMERS/NOB had a better degree of order than that of the HMERS. In addition, there was a slight increase in ΔHg, representing the shear resistance of starch particles at high temperatures, and this trend was more pronounced with increasing NOB contents (Table 2). The order degree of the rice starch–NOB complex was fundamentally improved under HME, which repressed the limiting of rice starch with α-amylase, and subsequently decreased the C_∞_ of rice starch. Even though NOB forms specific ordered domains that resist enzymatic hydrolysis by interacting non-covalently with starch molecules, it also separates during digestion and acts as an enzyme inhibitor. These two synergistic effects significantly enhance the anti-digestibility of rice starch.

## 4 Conclusion

The study investigated the combined effects of HME and NOB on the *in vitro* digestibility, thermal characteristics, multi-scale structures, and interactions of rice starch. HME was found to decrease both the anti-digestibility and thermal stability of rice starch. However, there are hydrophobic interactions and hydrogen bonding between NOB and starch, and the formation of complexes effectively hinders the digestion of NOB. This interaction disrupted the double helix structures of starch, favoring the formation of single helix structures. The synergistic modification of HME and NOB, rather than HME alone, resulted in an enhanced ordered structure of starch and a slight improvement in ΔHg. These findings highlight the combined influence of HME and NOB on the multi-scale structures and properties of rice starch. Overall, the study suggests that synergistic modification of HME and NOB could enhance the nutritional value and functional properties of rice starch, potentially broadening its high-value applications. However, the anti-digestibility of the complex raises questions about its prebiotic activity. Further research is needed to investigate the fermentation process and prebiotic potential of the complex systematically.

## Data availability statement

The original contributions presented in the study are included in the article/supplementary material, further inquiries can be directed to the corresponding author.

## Author contributions

ZZ: Data curation, Investigation, Methodology, Software, Writing – original draft. YF: Data curation, Investigation, Methodology, Software, Writing – review & editing. HW: Data curation, Formal analysis, Investigation, Methodology, Software, Writing – review & editing. HH: Funding acquisition, Methodology, Project administration, Resources, Supervision, Validation, Visualization, Writing – review & editing.
