# Supplementary material for: Synergistic modification of hot-melt extrusion and nobiletin on the multi-scale structures, interactions, thermal properties, and in vitro digestibility of rice starch
Source: Front Nutr. 2024 May 15;11:1398380. doi: 10.3389/fnut.2024.1398380 (PMC11133735; doi:10.3389/fnut.2024.1398380)
Supplement: Supplementary file 1 [file Image_1.pdf]

Supplementary Figures

Synergistic modification of hot-melt extrusion and nobiletin on the multi-scale structures, interactions, thermal properties, and *in vitro* digestibility of rice starch

Zhihong Zhang [1], Ying Feng [1], Honglan Wang [1], Hai He [1, 2, *]

[1] Department of Nutrition and Food Hygiene, School of Public Health, Heinz Mehlhorn Academician Workstation, Hainan Medical University, Haikou, Hainan, China,

[2] Department of Endocrinology and Metabolism, Shunde Hospital, Southern Medical University (The First People's Hospital of Shunde), Foshan, Guangdong, China

[*] **Corresponding author**

Contact: Hai He, No. 3 Academy Road, Longhua District, Haikou 571199, Hainan Province, China.

E-mail address: h.hai@hainmc.edu.cn.

Supplementary Figure 1

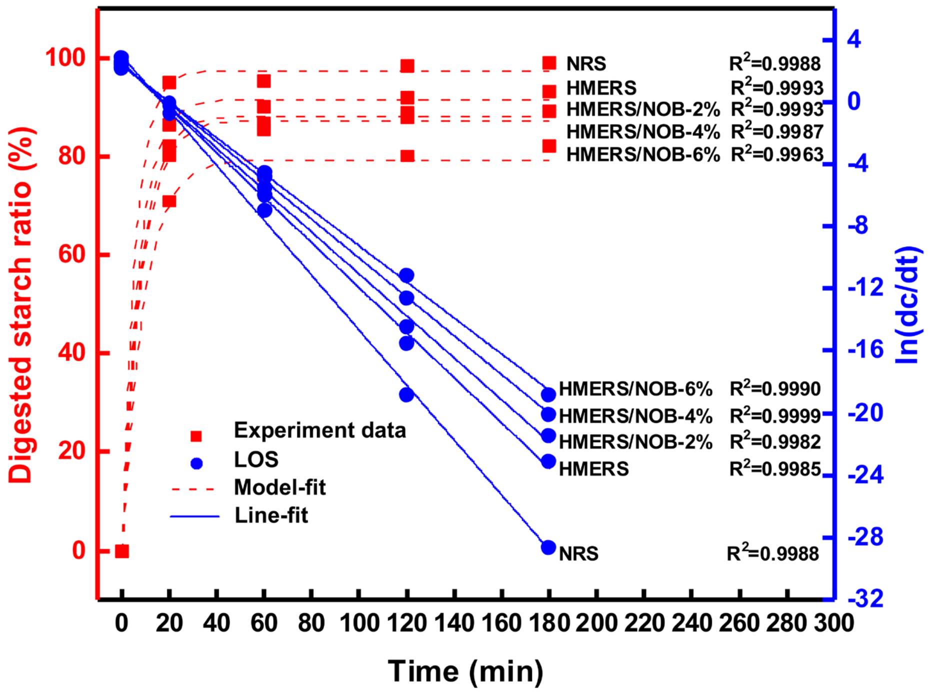

Supplementary Figure 1. Digestibility curves and LOS plots for NRS and HMERS samples. LOS, the logarithm of the slope; NRS, native rice starch; HMERS, hot-melt extruded rice starch; HMERS/NOB, hot-melt extruded rice starch–nobiletin complex.

Supplementary Figure 2

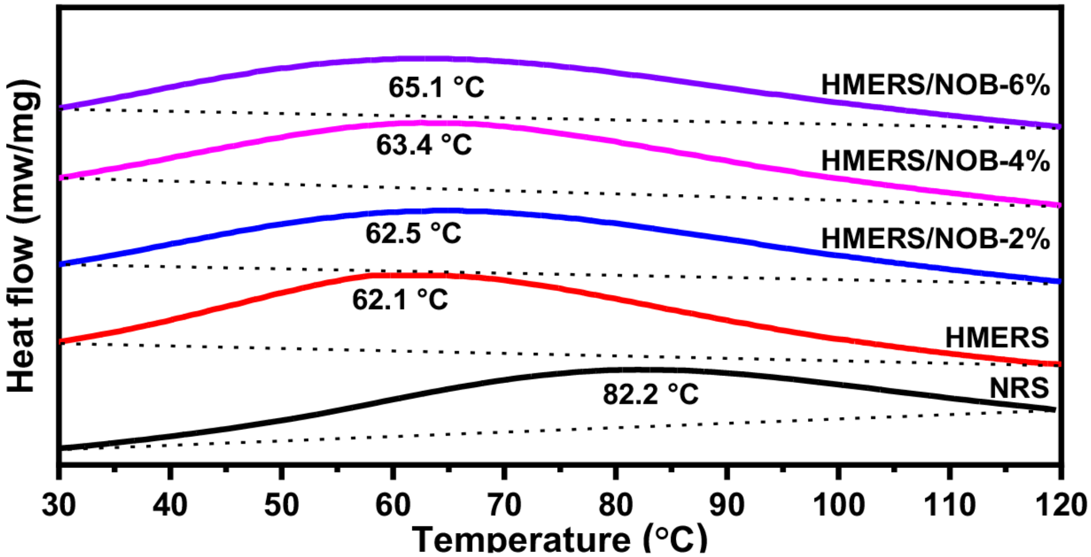

Supplementary Figure 2. DSC thermograms for NRS and HMERS samples. NRS, native rice starch; HMERS, hot-melt extruded rice starch; HMERS/NOB, hot-melt extruded rice starch–nobiletin complex.
